# Design and Analysis of a Polymeric Left Ventricular Simulator via Computational Modelling

**DOI:** 10.3390/biomimetics9050269

**Published:** 2024-04-28

**Authors:** Turgut Batuhan Baturalp, Selim Bozkurt

**Affiliations:** 1 Department of Mechanical Engineering, Texas Tech University, P.O. Box 41021, Lubbock, TX 79409, USA; 2 School of Engineering, Ulster University, York Street, Belfast BT15 1AP, UK

**Keywords:** left ventricle, left ventricular simulator, pneumatic artificial muscle

## Abstract

Preclinical testing of medical devices is an essential step in the product life cycle, whereas testing of cardiovascular implants requires specialised testbeds or numerical simulations using computer software Ansys 2016. Existing test setups used to evaluate physiological scenarios and test cardiac implants such as mock circulatory systems or isolated beating heart platforms are driven by sophisticated hardware which comes at a high cost or raises ethical concerns. On the other hand, computational methods used to simulate blood flow in the cardiovascular system may be simplified or computationally expensive. Therefore, there is a need for low-cost, relatively simple and efficient test beds that can provide realistic conditions to simulate physiological scenarios and evaluate cardiovascular devices. In this study, the concept design of a novel left ventricular simulator made of latex rubber and actuated by pneumatic artificial muscles is presented. The designed left ventricular simulator is geometrically similar to a native left ventricle, whereas the basal diameter and long axis length are within an anatomical range. Finite element simulations evaluating left ventricular twisting and shortening predicted that the designed left ventricular simulator rotates approximately 17 degrees at the apex and the long axis shortens around 11 mm. Experimental results showed that the twist angle is 18 degrees and the left ventricular simulator shortens 5 mm. Twist angles and long axis shortening as in a native left ventricle show it is capable of functioning like a native left ventricle and simulating a variety of scenarios, and therefore has the potential to be used as a test platform.

## 1. Introduction

Preclinical testing of medical devices is an essential step in the product life cycle [1], whereas testing of cardiovascular implants such as valve substitutes or continuous flow left ventricular assist devices requires specialised testbeds or numerical simulations using computer software [2,3]. For instance, a transcatheter aortic valve was tested considering ISO standards to evaluate clinical performance using a patient-specific in vitro test setup [4]. The hydrodynamic performance of a transcatheter mitral valve was evaluated using a commercial pulse duplicator [5]. Surgical and transcatheter pulmonary valve devices have also been tested using in vitro setups [6,7]. Specialised test setups have also been used to simulate clinical scenarios and left ventricular assist device support. For instance, Rocchi et al. studied suction incidents caused by a left ventricular assist device to validate clinical data using a test bench [8]. A novel continuous flow left ventricular assist device flow rate control algorithm to improve arterial pulsatility was tested using a mock circulatory system [9]. Test setups simulating cardiac function in these studies are actuated using pistons driven by complex systems and drivers; therefore, these setups are costly [10].

Ex vivo passive beating experimental cardiac models provide anatomical geometries in the testing of cardiovascular devices [11,12,13]. Heart valve function during left ventricular assist device support was evaluated using a passive beating experimental cardiac model [14]. Heart valve biomechanics can also be tested in these setups [15]. Moreover, measurements, such as chordae tendineae tension, which cannot be collected easily in vivo in the mitral valve apparatus can be collected using additional sensors in the ex vivo systems [16]. Nonetheless, passive beating heart platforms or ex vivo heart valve models also require actuators, which consist of pistons and complex drivers to generate the cardiac signals over an entire cardiac cycle. Therefore, although passive beating heart platforms provide advantages, such as working with anatomical structure, their actuation system has similar limitations to mock circulatory systems.

Isolated beating heart platforms do not need external actuators because the electrical activity of the heart is preserved during the experiments [17,18]. Isolated beating heart platforms can be used to evaluate a variety of scenarios, such as continuous flow left ventricular assist device support [19,20], heart valve performance [21], and myocardial perfusion [22] or they can be used as a training tool [23]. Nonetheless, experimental data show that a lack of autoregulatory mechanisms in isolated hearts may result in relatively high heart rates and continuous hyperemia [24,25].

Animal models provide realistic conditions for the clinical cases [26,27,28]. Therefore, animal models have been used to evaluate cardiovascular devices. For instance, sheep or bovine models were used to evaluate a centrifugal flow left ventricular assist device [29,30,31]. Animal models have also been used to develop and test prosthetic heart valve devices [32]. However, increasing ethical concerns and developments in computational models established roadmaps to refine, reduce, and replace animal experiments [33,34,35].

Lumped and distributed parameter models have been used in cardiovascular research to evaluate clinical cases and cardiovascular devices [36]. Lumped parameter models simulate blood flow rates and pressures in different compartments of the cardiovascular system; therefore, they are suitable for simulating the whole cardiovascular system over a relatively short period [37,38,39]. For instance, the evaluation of cardiac function and blood flow in the cardiovascular system during left ventricular assist device support and atrial fibrillation is presented in [40]. Nonetheless, lumped parameter models provide information only about pressures and blood flow rates in the cardiovascular system. Computational fluid dynamics models are used to simulate blood flow velocities and related parameters such as wall shear stresses in the cardiovascular system [41]. Computational fluid dynamics models have been used in heart valve and continuous flow left ventricular assist device research [42,43]. Although computational fluid dynamics simulations provide detailed information about the blood flow in an anatomical structure, they may be computationally expensive [44,45,46].

It is clear that there is a need for low-cost, relatively simple and efficient test beds that can provide realistic conditions to simulate physiological scenarios and evaluate cardiovascular devices. The aim of this study is to design and analyse a left ventricle simulator resembling native left ventricular geometry and simulating left ventricular wall motion in a realistic way.

## 2. Materials and Methods

**Figure 1:**
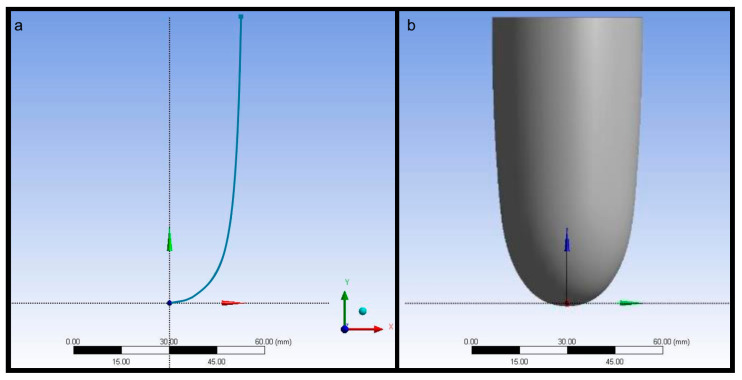
(**a**) The section curve used to generate the left ventricle wall and (**b**) the generated left ventricle geometry.

The shape and dimensions of the left ventricle chamber were selected considering anatomical ranges of basal diameter and long axis length in a healthy heart. The left ventricular basal diameter changes between 39 mm and 56 mm and the long axis length changes between 90 mm and 104 mm [47,48,49]. Therefore, the left ventricular basal diameter was 45 mm, whereas the long axis length was 97 mm in the geometric model. The diameter of the left ventricle model was reduced along the long axis to 40 mm and the apex of the left ventricle model was created using a radius curve tangent to the horizontal axis. The volume of the left ventricle model was 118 mL within the physiological end-diastolic left ventricle volume over a cardiac cycle [50,51]. A line geometry revolved around the vertical axis to create a 1 mm thick left ventricle chamber. The thickness of the left ventricle model was decided considering the mechanical properties of latex rubber [52]. The section curve used to generate the left ventricle wall and the generated left ventricle geometry are given in Figure 1.

Pneumatic artificial muscles have been proposed to activate ventricular contraction in cardiac simulators [53]. In this study, a similar activation method was utilised using helical tubes around the left ventricle chamber [54]. The pneumatic artificial muscles in the actual beating left ventricle simulator were positioned in helical orientation to increase the pumping performance with the help of torsional contraction and to mimic the swirling pattern of the cardiac muscles. The general assembly pattern has a 90-degree shift between two ends of the muscles to make installing pneumatic artificial muscles easier. Since projection on two-dimensional space is already a quarter circle, it creates a helical curve when it is projected to a three-dimensional space. However, a helical curve should have conical features to follow the conic geometry of the geometric model. Therefore, a conical spiral was chosen to generate a spiral arrangement of muscles. The general form of parametric equations is given as follows for a conical spiral.

(1) x=t×r×cos(a×t)

(2) y=t×r×sin(a×t)

(3) z=t

**Figure 2:**
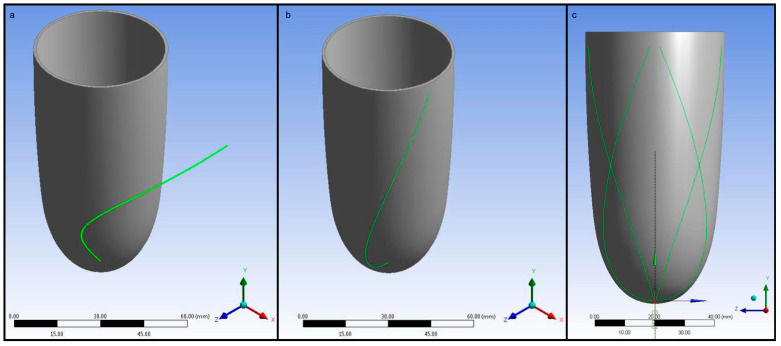
(**a**) The input conical spiral in Ansys Design Modeler, (**b**) a projected curve, and (**c**) the guidelines to create pneumatic artificial muscle geometries.

Here, *a* is the angular frequency, *z* is the height of the cone, and *r* is the radius. The angular range was chosen as 0 to π/2 radians for a 90-degree shift. MATLAB 2016b (Mathworks, Natick, MA, USA) was used to generate a curve for the conical spiral and as an input file in ANSYS 2016 Design Modeler (ANSYS, Canonsburg, PA, USA). A three-dimensional line body is generated in ANSYS Design Modeler using the generated curve in MATLAB 2016b (Mathworks, Natick, MA, USA). The conical curve was projected onto the geometric models as described above using the projected paths as a guideline for actuator generation. Four pneumatic artificial muscles were generated around the left ventricle chamber so as not to make the left ventricle chamber too stiff during the ejection phase [54]. The input conical spiral in Ansys Design Modeler, a projected curve, and the guidelines to create pneumatic artificial muscle geometries are given in Figure 2.

**Figure 3:**
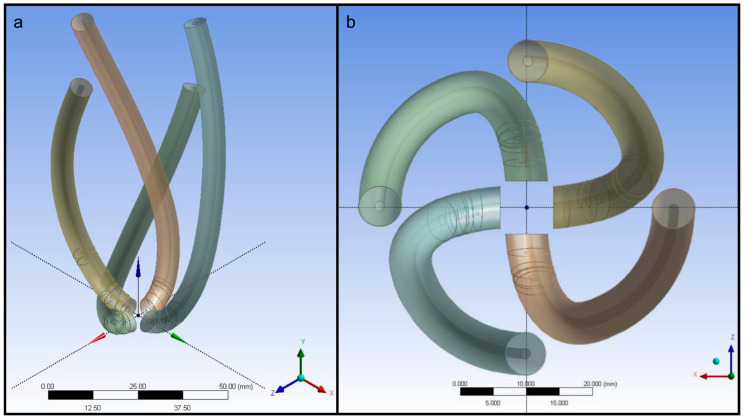
The pneumatic artificial muscles: (**a**) isometric view, (**b**) top view.

The pneumatic artificial muscle geometries were generated using the guideline curves utilising Sweep operation in ANSYS Design Modeler. The diameter of the pneumatic artificial muscles was 5 mm in the finite element model. The pneumatic artificial muscles from the isometric and top views are given in Figure 3.

**Figure 4:**
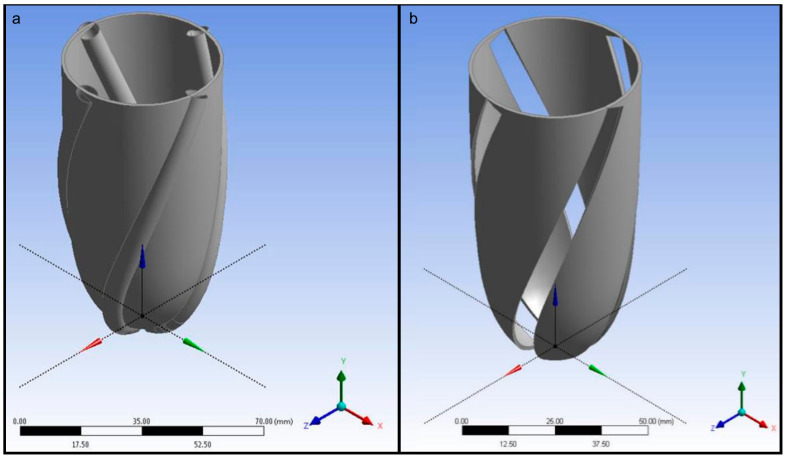
Left ventricle chamber (**a**) with and (**b**) without pneumatic artificial muscles.

The generated pneumatic artificial muscle geometries were subtracted from the left ventricle geometry to remove the material in the left ventricle walls where the pneumatic artificial muscles are positioned. The geometry of the left ventricle chamber with and without pneumatic artificial muscles is given in Figure 4.

**Figure 5:**
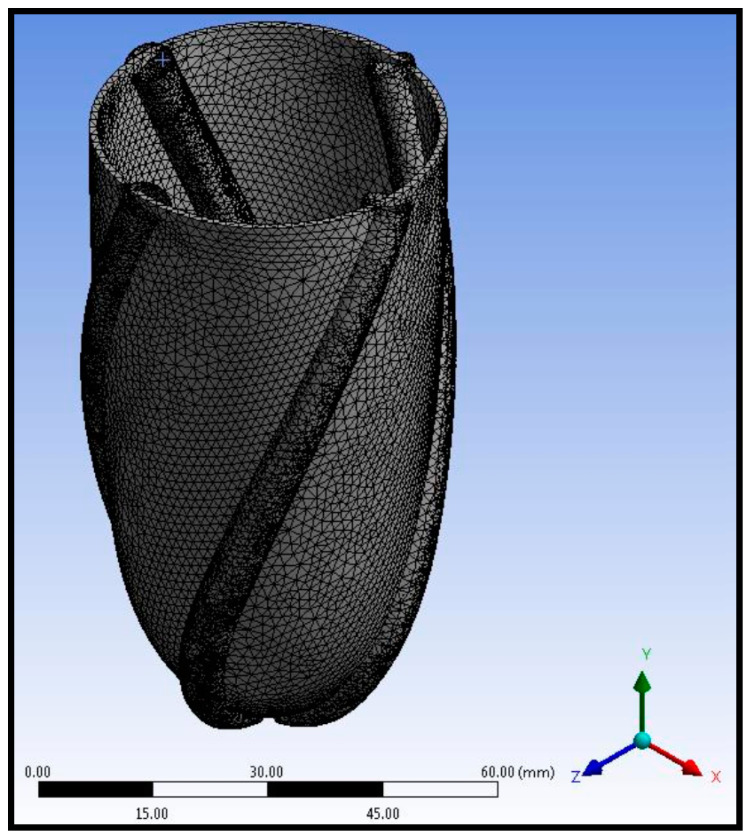
The meshed left ventricular simulator body with ventricle wall and pneumatic artificial muscles.

The complete model with left ventricle walls and pneumatic artificial muscles was composed of meshed tetrahedral elements. The number of elements in total was around 118,000 after the grid independence test. Pneumatic artificial muscles and ventricle walls were connected using bonded contact. The meshed left ventricular simulator body is given in Figure 5.

Linear elastic and isotropic material models for the left ventricle wall and pneumatic actuated muscles were used in the simulations. Latex rubber was considered in the left ventricle wall. Therefore, the Elastic Modulus and Poisson’s Ratio of the left ventricle walls were 68.9 kPa and 0.499, whereas the Elastic Modulus of the pneumatic artificial muscles was 1.78 MPa [55] and Poisson’s Ratio of the pneumatic artificial muscles was 0.35 [56].

Contraction of the left ventricular simulator was simulated using thermal expansion in the pneumatic artificial muscles. The thermal expansion coefficient of the pneumatic artificial muscles was acquired by an experimental procedure to match the shrinking behaviour of the finite element model of the pneumatic artificial muscle with air pressure expansion, which simulates the cardiac contraction [52]. The equation below was used to determine the thermal expansion coefficient:

(4) ΔL=α×L×ΔT

Here, Δ*L* is the change in the length of a pneumatic artificial muscle, *L* is the initial length of a pneumatic artificial muscle, Δ*T* is the change in temperature, and *α* is the thermal expansion coefficient. The contraction of the ventricle model is driven by the Δ*T* term and the coefficient α. Because the Δ*T* term is fixed, it is required to find the correct values of α to achieve realistic results. An arbitrary Δ*T* of 1000 degrees Celsius is chosen and the thermal expansion coefficient was determined to be 14.36 × 10^−5^ 1/°C in the finite element model.

**Figure 6:**
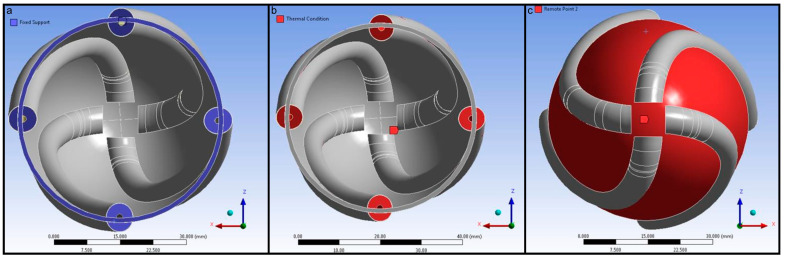
Boundary conditions used in the geometry of the left ventricular simulator: (**a**) fixed support at the upper side of the left ventricle and pneumatic artificial muscles, (**b**) thermal condition in the pneumatic artificial muscles, (**c**) remote point at the apex of the left ventricle and the outer surface of the left ventricle wall.

A fixed support boundary condition was applied at the base of the left ventricle and the pneumatic artificial muscles considering that the left ventricular simulator will be installed on an experimental setup at the base. The pneumatic artificial muscles were loaded with a thermal condition as described above. Torsional or twisting movement is very important to generate a real left ventricle-like wall motion. Therefore, the “Remote Points” tab was used at the apex of the left ventricle and the outer surface of the left ventricle wall. Boundary conditions used in the geometry of the left ventricular simulator are given in Figure 6.

Prototypes for the left ventricle simulator were built using mould-making latex rubber from AeroMarine Products Inc. (San Diego, CA, USA). Contractions in the left ventricle simulator were actuated using pneumatic artificial muscles, including flexible inner tubes and the braided sleeves. Pneumatic artificial muscles were pressurised using a three-way two-position-type pneumatic electric solenoid to activate the left ventricle chamber over each cycle. The prototyped left ventricle models were fixed on a holder at their base, as simulated in the finite element analyses. The air pressure was set to 80 psi, because higher air pressures in the pneumatic artificial muscles caused damage to ventricle walls made of latex rubber.

## 3. Results

**Figure 7:**
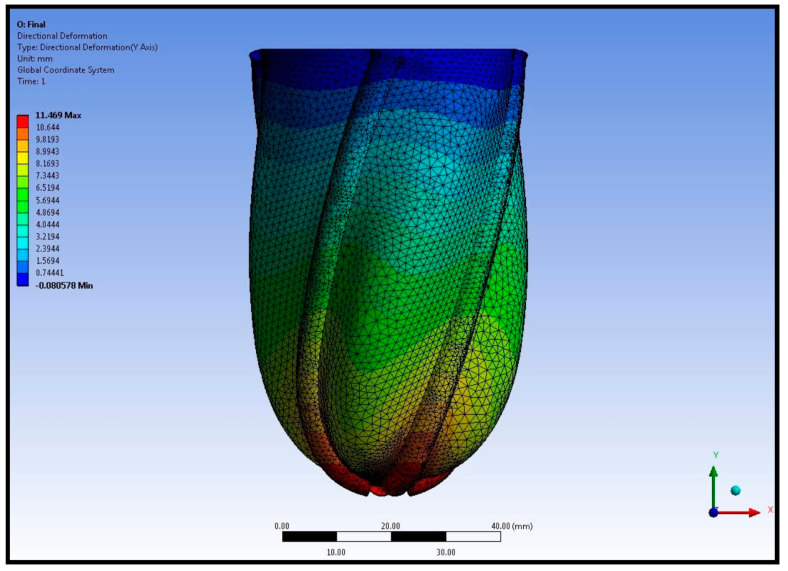
Shortening of the left ventricular long axis in the left ventricular simulator.

The left ventricular contraction was evaluated considering the shortening of the left ventricular long axis and torsion of the pneumatic artificial muscles. The shortening of the left ventricular long axis is given in Figure 7.

**Figure 8:**
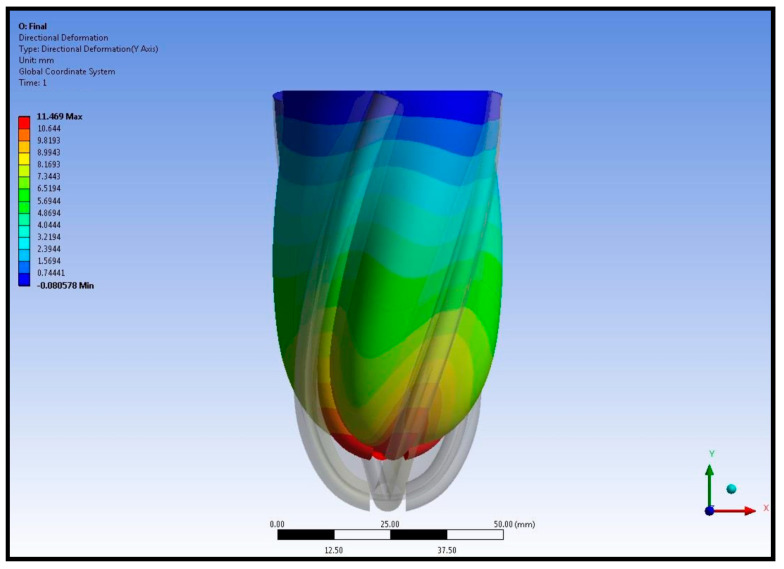
Comparison between the original left ventricular geometry and the deformed left ventricular geometry.

Maximal left ventricular shortening was around 11 mm at the apex of the left ventricle. The shortening of the left ventricle around the left ventricular mid-axis was nearly 5 mm. A comparison between the original left ventricular geometry and the deformed left ventricular geometry is given in Figure 8.

**Figure 9:**
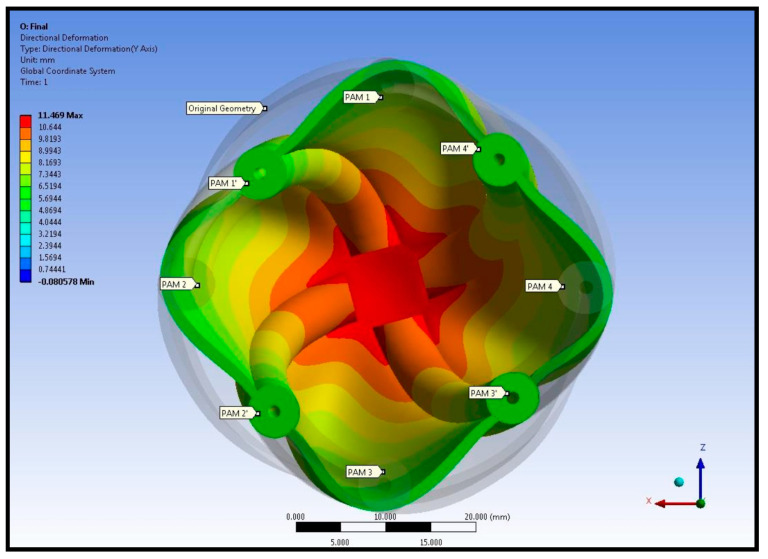
Comparison of original and deformed left ventricle geometries at the left ventricular mid-section from the top view.

The displacement of the left ventricular wall and pneumatic artificial muscles is shown in Figure 8. Contraction of the pneumatic artificial muscles shortens the left ventricle at the apex. A comparison of original and deformed left ventricle geometries at the left ventricular mid-section from the top view is given in Figure 9.

**Figure 10:**
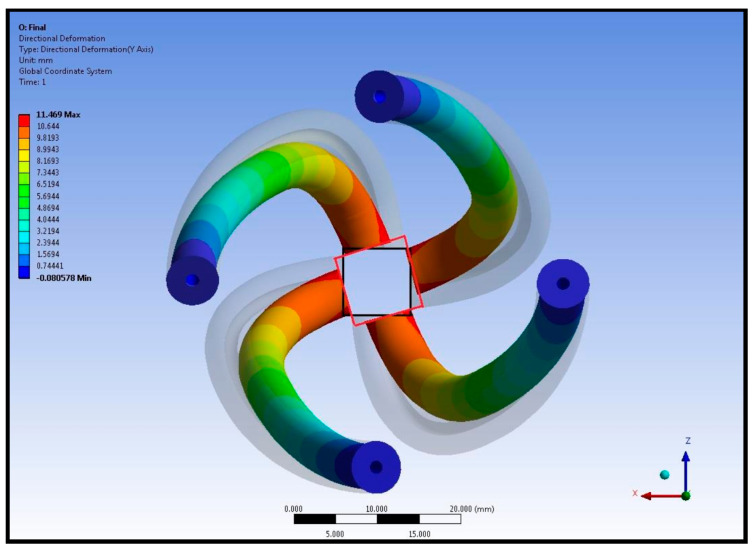
Comparison of the displacement and rotation in the pneumatic artificial muscles in the free and deformed left ventricular model.

Results in Figure 9 show the left ventricular torsion. The orientation of pneumatic artificial muscles was altered by twisting in a counterclockwise direction. Moreover, the shape of the elastomer body is also altered. Because the elastomer body is not thermally loaded, deformation on the body should be a consequence of thermal loading, in other words, contraction of pneumatic artificial muscles. Displacement and rotation in the pneumatic artificial muscles together with a comparison of relaxed pneumatic artificial muscles are given in Figure 10.

**Figure 11:**
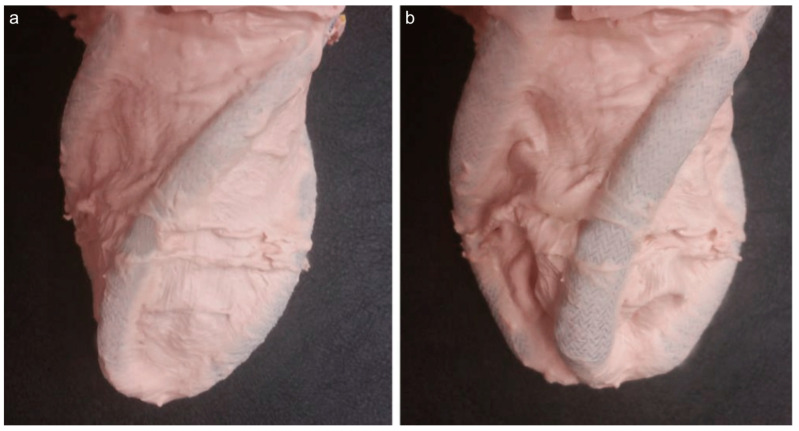
Side view of a prototype of the left ventricular chamber: (**a**) free left ventricular geometry (**b**), deformed left ventricular geometry due to pressurised pneumatic artificial muscles.

The results show how pneumatic artificial muscles undergo deformation, whereas the transparent silhouette shows the original pneumatic artificial muscle shapes. The unloaded pneumatic artificial muscle orientation creates a hypothetical rectangle in the apex region. In addition to the initial rectangle shown in black in Figure 10, the red rectangle depicts the hypothetical rectangle in between the deformed apex. The twist angle of the apex of the left ventricle model was calculated by using the defined triangles. Torsional movement is essential to generate actual human left ventricle-like wall motion and its existence is ensured. Finite element analysis predicted a rotation at the apex of around 17 degrees and an average rotation in the elastomer body at around 7 degrees. A side view of a prototyped left ventricular chamber in free and deformed configurations during a dry run is given in Figure 11.

**Figure 12:**
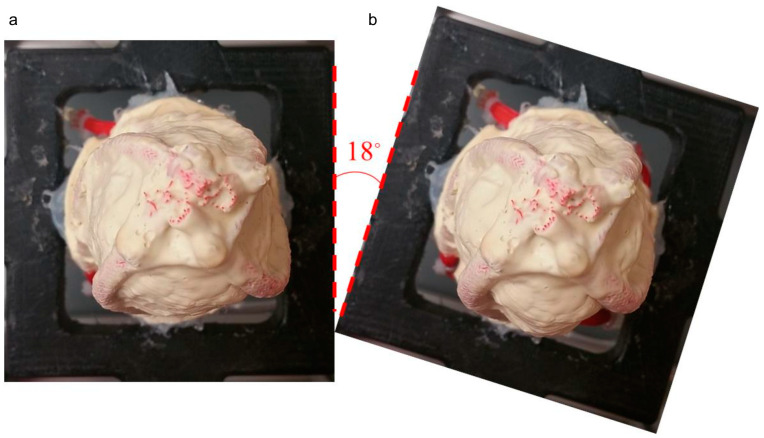
Inferior view of a prototype of the left ventricular chamber: (**a**) free left ventricular geometry, (**b**) deformed left ventricular geometry due to pressurised pneumatic artificial muscles.

Pressurised pneumatic artificial muscles shorten and twist the left ventricular chamber. The shortening of the left ventricle chamber was around 5 mm. The twist angle in a prototype of the left ventricle model during a dry run is given in Figure 12.

The twist angle in the left ventricle model was calculated by rotating the image of the deformed geometry until the orientation of the pneumatic artificial muscles matched with the original undeformed geometry. Twist angle in the left ventricle chamber was around 18 degrees.

## 4. Discussion

In this study, a novel left ventricular simulator was designed and finite element simulations were performed to evaluate the contractile behaviour of the developed left ventricular simulator. Static structural and thermal simulations were performed to understand the deformation in the left ventricle wall made of latex rubber. Also, prototype production of the proposed left ventricular simulator was carried out to evaluate the validity of the simulations. The developed system is activated by four pneumatic artificial muscles by simply pressurising them using air. A solenoid can control the airflow in the system. Moreover, torsional movement of the left ventricular wall can be replicated because of the orientation of the pneumatic artificial muscles in the system. The proposed left ventricular simulator can be manufactured using simple moulds and commercially available polymeric materials such as latex rubber, as shown in this study. Therefore, the developed system is affordable and easy to manufacture and drive, unlike the sophisticated pulse duplicators, which require high-cost hardware to operate the system [10].

The motion of the left ventricle wall is crucial for the effective pumping function of the left ventricle. Moreover, the torsional movement of the left ventricle is achieved via the helical orientation of myocardial fibres [57,58,59,60]. The simulations revealed that the contractile behaviour of the designed left ventricular simulator can imitate the native left ventricular wall deformations in a contracted state. A realistic wall motion in the designed left ventricular simulator was also verified by the twisting angle at the apex. Apical to the basal rotation angle of a healthy ventricle is around 16 degrees [61], whereas maximal long axis shortening in a healthy ventricle is around 10 mm [47]. The simulation results showed that the designed left ventricular simulator rotates around 17 degrees at the apex and shortens 11 mm. Moreover, experimental results show that the twist angle and apex shortening are around 18 degrees and 5 mm. Therefore, the wall motion and torsion in the developed left ventricular simulator are comparable to a healthy ventricle.

Torsional deformation in the native left ventricle is a result of fibre orientation in the ventricle wall [62,63]. Helical flow patterns in the aorta are generated by the torsional deformation of the left ventricular wall and aortic valve angle [64]. The developed left ventricular simulator may generate helical flow patterns at the outlet of the left ventricle because of its design. Flow patterns in aortic valve substitutes may cause complications such as thrombus formation and preclinical testing of these devices requires specialised techniques such as particle image velocimetry [65,66]. However, due to the operating principles of the pulse duplicator, the generated flow patterns in the left ventricle and left ventricular outlet site may not be realistic. Therefore, the developed system in this study may help overcome the challenges of testing the heart valve substitutes before clinical applications.

Previously, cardiac assist devices driven by pneumatic artificial muscles were developed. Roche et al. [67] proposed a soft robotic sleeve driven by pneumatic artificial muscles. Despite the actuator design being somewhat similar to the designed left ventricular simulator, the primary aim of the designed platform in this study is to simulate cardiac function. Lorenzon et al. [68] proposed a left ventricle pump driven by inverse pneumatic artificial muscles. In this design, the inverse pneumatic artificial muscles drove the left ventricle with external helical contraction. In the developed system in this study, the pneumatic artificial muscles were embedded in the left ventricular wall to contract the left ventricle model instead of squeezing it.

The designed ventricular simulator has the following advantages over the existing mock circulatory systems: haemodynamic signals in pulse duplicators generated by piston motion [69,70]. Therefore, physiological effects such as septal shift during mechanical circulatory support may not be simulated in these systems [71,72,73,74]. For instance, a developed mock circulatory system includes both ventricles in a series connection driven by piston pumps [75]. Therefore, it can only simulate haemodynamic signals during left ventricular assist device support. However, the designed left ventricular simulator is capable of simulating left ventricular wall motion because it is made of a flexible material. This may be particularly useful for developing next-generation left ventricular assist devices or testing different configurations during mechanical circulatory support in clinical cases such as cardiogenic shock [76,77]. Beating heart platforms may provide realistic test conditions to evaluate physiological cases [78,79]. However, the use of animal models and animal tissue in research raises ethical concerns [80,81,82,83]. The designed left ventricular simulator may help reduce the use of animal models in experiments because the geometry of the left ventricle can be made anatomical and it can replicate left ventricular wall motion.

Dilated and hypertrophic cardiomyopathies are major causes of functional and anatomical remodelling in the left ventricle [84,85,86]. Dilated cardiomyopathy causes the left ventricle to enlarge, whereas the left ventricular muscle is thinned [87,88,89,90]. Hypertrophic cardiomyopathy may cause the left ventricular muscle to thicken [91,92,93]. Prototyping the left ventricular chambers in different sizes and thicknesses will allow for the simulation of dilated and hypertrophic cardiomyopathies, whereas traditional mock circulatory systems can only simulate left ventricular function. Moreover, left ventricular torsion may increase in the elderly and heart failure results in abnormal left ventricular mechanics [94,95,96]. All these effects can be simulated by modifying pneumatic artificial muscles in the developed left ventricular simulator. Other potential applications in this system also include testing of heart valve replacement implants where an anatomical shape will result in more accurate tests [97,98]. Patient-specific left ventricular models can also be manufactured and may provide benefits for cohorts such as paediatric patients. Diagnostic criteria in paediatric patients for heart failure are adapted from adults because of the limited available information for children [99,100]. The designed left ventricular simulator can simulate paediatric ventricles and may help to study clinical conditions in this cohort. Moreover, it may be possible to develop personalised models, which allow us to work on personalised patient-specific solutions for patients. However, it should be noted that this study presents the design approach and the concept of the left ventricular simulator.

The developed and tested left ventricular simulator made of flexible latex rubber also has potential for educational and training purposes in the field of cardiovascular medicine and biomedical engineering. The proposed setup may offer a tangible representation of cardiac anatomy and physiology unlike numerical models, which may be inaccessible or challenging to interpret for students and clinicians without a strong background in mathematics and computational science [101]. Moreover, students can gain practical insights into cardiac mechanics, haemodynamics, and pathology, resulting in a deeper understanding of cardiovascular concepts with experimentation with a physical device which can simulate myocardial motion. Current educational test platforms include passive beating heart platforms for testing, training, and teaching transcatheter therapies [13] or 3D-printed models [102]. The proposed model has the advantages of reducing the use of animal tissue and being flexible compared to the 3D-printed rigid models.

This study has a number of limitations. Hydrodynamic tests were not performed, and only left ventricular wall motion during contraction was analysed. Therefore, the hydrodynamic performance of the developed system remains to be studied. A more complete system including the right ventricle will allow for the study of the interaction between the ventricles. The septal shift is a concern for both ventricles during the continuous flow of left ventricular assist device support and may cause right ventricular failure, as mentioned above. Therefore, a more complete cardiac simulator including left and right ventricles will be manufactured in the future. Also, physiological and clinical scenarios such as cardiomyopathies will be considered in the future. Here, it should be noted that the aim of the study is to analyse and understand the design parameters using finite element simulations and show a proof of concept, manufacturing the prototypes of the proposed left ventricular simulator.

## 5. Conclusions

In this study, design concepts and finite element simulations are presented for a novel left ventricular simulator made of latex rubber. The simulation and experimental test results show that the designed left ventricular simulator can simulate left ventricular twists and shortening as in a native left ventricle. It combines the advantages of being affordable and manufacturability, the potential to generate realistic flow patterns, and practical applications to use as test and educational tools. It may also help to reduce the use of animal models, which raise ethical concerns. Therefore, it may replace or help to improve the existing test platforms being used for research and education.

## Data Availability

No new data were created or analysed in this study.
